# The ILEI/LIFR complex induces EMT via the Akt and ERK pathways in renal interstitial fibrosis

**DOI:** 10.1186/s12967-022-03265-2

**Published:** 2022-01-29

**Authors:** Jieqing Zhou, Hong Jiang, Hongkun Jiang, Yan Fan, Jing Zhang, Xiaoxue Ma, Xuewei Yang, Yu Sun, Xing Zhao

**Affiliations:** Department of Pediatrics, First Hospital of China Medical University, Shenyang, 110001 China

**Keywords:** Renal interstitial fibrosis (RIF), Epithelial-to-mesenchymal transition (EMT), Interleukin-like epithelial-to-mesenchymal transition inducer (ILEI), Leukemia inhibitory factor receptor (LIFR)

## Abstract

**Background:**

Chronic kidney disease (CKD) is characterized by high morbidity and mortality and is difficult to cure. Renal interstitial fibrosis (RIF) is a major determinant of, and commonly occurs within, CKD progression. Epithelial mesenchymal transition (EMT) has been identified as a crucial process in triggering renal interstitial fibrosis (RIF). Interleukin‐like EMT inducer (ILEI) is an important promotor of EMT; this study aims to elucidate the mechanisms involved**.**

**Methods:**

Male C57BL6/J mouse were randomly divided into 6 groups: sham (n = 10), sham with negative control (NC) shRNA (sham + NC, n = 10), sham with ILEI shRNA (sham + shILEI, n = 10), unilateral ureteral obstruction (UUO, n = 10), UUO with NC (UUO + NC, n = 10) and UUO with ILEI shRNA (UUO + shILEI, n = 10). Hematoxylin and eosin (H&E), Masson, and immunohistochemical (IHC) staining and western blotting (WB) were performed on murine kidney tissue to identify the function and mechanism of ILEI in RIF. In vitro, ILEI was overexpressed to induce EMT in HK2 cells and analyzed via transwell, WB, real-time PCR, and co-immunoprecipitation. Finally, tissue from 12 pediatric CKD patients (seven with RIF and five without RIF) were studied with H&E, Masson, and IHC staining.

**Results:**

Our in vitro model revealed that ILEI facilitates RIF in the UUO model via the Akt and ERK pathways. Further experiments in vivo and in vitro revealed that ILEI promotes renal tubular EMT by binding and activating leukemia inhibitory factor receptor (LIFR), in which phosphorylation of Akt and ERK is involved. We further find markedly increased expression levels of ILEI and LIFR in kidneys from pediatric CKD patients with RIF.

**Conclusion:**

Our results indicate that ILEI may be a useful biomarker for renal fibrosis and a potential therapeutic target for modulating RIF.

## Introduction

Chronic kidney disease (CKD) is a public health problem with increasing global prevalence (about 10–13% of the population [1, 2]). CKD can cause irreversible progressive loss of kidney function. CKD is usually asymptomatic until later stages when it is difficult to reverse, at which point end-stage renal disease (ESRD) often occurs. Therefore, CKD causes high disability and mortality. Renal interstitial fibrosis (RIF) is a common phenomenon in CKD and ESRD which closely relates to loss of renal function and motility. RIF is characterized by tubular atrophy, myofibroblast activation and proliferation, and extracellular matrix deposits [3]. Accumulating evidence indicates that epithelial cells play major roles in RIF via epithelial-to-mesenchymal transition (EMT). Following EMT, renal tubular epithelial cells (TECs) lose normal morphology, tight cell–cell junctions, and epithelial cell markers such as E‐cadherin, instead gaining mesenchymal markers including α‐smooth muscle actin (α‐SMA) and vimentin [4, 5]. Multiple cytokines regulate EMT, such as transforming growth factor beta 1 (TGF‐β1), which is known to induce both EMT and RIF [6, 7].

Interleukin-like EMT inducer (ILEI), a member of the family with sequence similarity 3 (FAM3) family, is widely expressed in human and mouse tissues and plays important roles in a variety of biological processes. It was originally discovered as a pivotal gene for Alzheimer’s disease [8] and is also a key factor in retinal formation [9, 10]. In the past 20 years, studies have found that ILEI plays important roles in EMT to promote formation, invasion, and metastasis in melanoma and breast, colon, prostate, lung, and liver cancers [11–13]. Our previous research demonstrates that ILEI is involved in TGF‐β1-induced renal tubular EMT, with ILEI overexpression independently driving EMT in HK‐2 cells and enhancing EMT in response to TGF‐β1 through the Akt and ERK pathways [14].

The leukemia inhibitory factor receptor (LIFR) acts as a signaling platform for a variety of cytokines. It is involved in maintaining hepatocyte pluripotency, protecting the liver, promoting glucose absorption and utilization, regulating cellular proliferation and differentiation, and other biological processes. Studies have reported that LIFR activates the JAK/STAT, ERK/MAPK, and Akt/PI3K pathways in tumor formation, inflammation, and cardiac function [15–17]. Woosley, et al. found that ILEI forms a ligand-receptor complex by binding to the extracellular binding factor region of LIFR [18], suggesting that LIFR may be a key downstream effector molecule for many functions of ILEI. However, no research defines interactions of ILEI and LIFR in RIF.

In a previous study, we demonstrated that ILEI likely mediates TGF‐β1–induced EMT via the Akt and ERK pathways [14]. However, the specific mechanisms through which ILEI promotes EMT during RIF progression are undefined. Here, we test whether LIFR participates in ILEI-induced EMT and whether this contributes to RIF formation. We explore possible mechanisms in vitro then use mouse and human fibrotic kidney tissue to verify the results in vivo.

## Materials and methods

### Experimental animals and antibodies

Male C57BL6/J mice weighing 20–25 g were housed at the Ethics Committee for Animal Experiments of the First Hospital of China Medical University under a 12 h:12 h light/dark cycle with a constant temperature (22 ± 1 °C) and free access to food and water. All animal experiments were performed according to the National Institutes of Health guide for the care and use of Laboratory animals, and were approved by the Animal Care and Use Committee of the First Hospital of China Medical University.

The detailed information of antibodies are as follows: Anti-ILEI antibody (ab72182, Abcam, Cambridge, U.K.), Anti-α‐SMA antibody (ab5694, Abcam), Anti-vimentin antibody (ab137321, Abcam), Anti-E‐cadherin antibody (ab133597, Abcam), Anti-p‐Akt antibody (ab18206, Abcam), Anti-Akt antibody (ab126811, Abcam), Anti-p‐ERK antibody (ab223500, Abcam), Anti- ERK antibody (ab17942, Abcam), Anti-collagen Ι antibody (ab34710, Abcam), Anti-LIFR antibody (sc-659, Santa cruz, Dallas, U.S.A.),Anti-collagen ΙII antibody (WL03186,wanleibio,Shenyang, China), Anti-β‐actin antibody (WL01845,wanleibio).

### Establishing the UUO mouse model and ILEI specific shRNA treatment

Mice were randomly divided into six groups: sham (n = 10); sham + NC (n = 10); sham + shILEI (n = 10); UUO (n = 10); UUO + NC (n = 10); UUO + shILEI (n = 10).

After anesthesia, the left kidney and the left ureter of mice were exposed, 100 µl virus solution (AAV9, 1 × 10^11^ viral genome particles) was retrogradely infused into the ureter according to group setup. And then they were subjected to UUO or sham operation, the UUO procedure was performed as previously described [19]. Mice were sacrificed 14 days after operation, and the kidneys were harvested, stored in liquid nitrogen or fixed with paraformaldehyde solution (4%) for various analyses.

### Histology and immunohistochemistry assays

The kidney samples fixed in 4% paraformaldehyde were embedded in paraffin, sectioned at a thickness of 5 μm, then stained using hematoxylin and eosin (H&E) and Masson staining.

For IHC analysis, paraffin-embedded kidney sections were deparaffinized in xylene, hydrated in graded alcohol and water, then placed in 3% H_2_O_2_ to eliminate endogenous peroxidase activity. Sections were then incubated with primary antibodies overnight. Images were captured using an OLYMPUS DP73.

### Cells culture and morphological observation

The human proximal tubular epithelial cell line (HK2) was obtained from China Center for Type Culture Collection HK2 cells were cultured in DMEM (12100-46, Gibco, Gaithersburg, U.S.A) with 10% FBS (FSS500, ExCell Bio, Shanghai, China) at 37 °C with 5% CO_2_. Cellular morphology was observed under a phase‐contrast microscope (OLYMPUS IX53).

### Grouping and transfection

To overexpress ILEI, pcDNA3.1-ILEI constructs were identified, sequenced and purchased by the Shanghai Biological Engineering Company. Three shRNA molecules targeting the LIFR gene and its negative control (NC) shRNA were designed and synthesized by GenePharma (Shanghai, China). HK-2 cells were transfected with indicated vector or shRNA using the Lipofectamine 2000 corresponding to the manufacturer’s instruction. After 48 h of transfection, cells were harvested to analyze transfection efficiency by Real-time PCR and Western blot. HK-2 cells were co-transfected with ILEI overexpression vector and the most effective LIFR shRNA or corresponding negative control (NC), cells were divided into four groups: empty vector, ILEI-OE (ILEI overexpression), ILEI-OE + NC, and ILEI-OE + shLIFR groups. Follow-up tests were performed 48 h after transfection. The primer sequences of all shRNA are listed in Table 1.

**Table 1:** Primer sequence of shRNA

shRNA	Sequence (5′–3′)
shRNA1	GGGGCTCCTCATGATTTGAAGTTTCAAGAGAACTTCAAATCATGAGGAGCCCTTTTT
shRNA2	GGCTGGATATCCACCAGATACTTTCAAGAGAAGTATCTGGTGGATATCCAGCTTTTT
shRNA3	GGGAAGACATTCCTGTGGAAGATTCAAGAGATCTTCCACAGGAATGTCTTCCTTTTT
NC	GCTTCTCCGAACGTGTCACGTTTCAAGAGAACGTGACACGTTCGGAGAAGTTTTT

### Transwell assays

Migration ability was evaluated in transfected HK2 cells via transwell migration assays. Briefly, 4 × 10^3^ cells from each group were seeded into the upper chamber of a transwell (3422, Corning, NY, U.S.A.) in 200 μl of serum‐free medium, with 800 μl of complete medium added to the lower chamber. All cells were then incubated for 24 h at 37 °C with 5% CO_2_. Cells which migrated through the membrane were stained with crystal violet (0528, Amresco, Solon, U.S.A.) and photographed and counted using an inverted microscope. Five randomly selected fields were quantified to find an average number.

### RNA isolation and real-time PCR

Total RNA was extracted using the TRIpure reagent (RP1001, BioTeke, Beijing, China). Poly-A tails were added to miRNA using the Poly (A) Tailing Kit (AM1350, Ambion, Waltham, MA, U.S.A.). The PrimeScriptTM RT reagent kit with gDNA Eraser (RR047B, Takara, Dalian, China), and gene-specific or random primers were used to generate cDNA. Real-time PCR was performed in an ExicyclerTM 96 Real-Time PCR system (BIONEER, Korea) using SYBR Green (SY1020, Solarbio, Beijing, China). The 2^−△△ CT^ method was used to calculate relative RNA expression levels from real-time fluorescence quantitative analysis [20]. Oligonucleotide primer sets are shown in Table 2.

**Table 2:** Oligonucleotide primer sets for polymerase chain reaction

Name	Forward sequence (5′–3′)	Reverse sequence (5′–3′)	Product size (bp)
LIFR	ACATCATCAGCGTAGTGGC	TTCCGACCGAGACGAGTTA	197
ILEI	TGAAGGCCATACAAGATG	CACCACAGAAGACCCAGT	153
E-cadherin	GAACGCATTGCCACATACAC	TGGTGTAAGCGATGGCGGCA	244
α‐SMA	GGGGTGATGGTGGGAATG	GCAGGGTGGGATGCTCTT	190
Vimentin	GACAGGCTTTAGCGAGTTATT	ACCGTTAGACCAGATTGATTC	160
β‐Actin	CACTGTGCCCATCTACGAGG	TAATGTCACGCACGATTTCC	155

*LIFR* leukemia inhibitory factor receptor, *ILEI* interleukin-Like epithelial-mesenchymal transition inducer, *α‐SMA* α-smooth muscle actin

### Western blotting

Cells or kidney tissue were lysed in lysis buffer containing proteinase inhibitors (WLA019,Beyotime, Shanghai, China) on ice, followed by centrifugation for 10 min at 4 °C 12,000 rpm, then the protein concentration was assessed via BCA protein assay kit (WLA004,Beyotime). Samples were separated by SDS-PAGE followed by transferring to polyvinylidene difluoride membranes (R1DB89779, Sigma-Aldrich, Millipore, U.S.A.) and blocked with 5% fat-free milk at roofHYm temperature for 1 h. After an overnight incubation at 4 °C with primary antibodies, the membranes were incubated with secondary antibodies for 1 h at 37 °C. Immunoreactive bands were visualized by enhanced chemiluminescence (7Sea Biotech). Band intensities were quantified using Gel‐Pro Analyzer 4, with β‐actin as an endogenous reference.

### Co-immunoprecipitation(Co-IP)

The endogenous interaction between ILEI and LIFR in HK-2 cells was verified by co-IP assay. The cell lysates of HK-2 cells in different groups were incubated with protein A-agarose beads and anti-ILEI or anti-LIFR antibody at 4 °C. Then the immunoprecipitated proteins were subjected to immunoblotting with anti-LIFR and anti-ILEI antibody.

### Patients and samples

12 pediatric patients with CKD who underwent renal biopsy between February 2017 and May 2021 at the First Hospital of China Medical University were included, of which 7 were confirmed to have RIF and 5 were negative for RIF. Among the 7 patients in the RIF group, 3 cases had Lupus nephritis and 3 had Henoch-Schonlein purpura nephritis and 1 case had microscopic polyangiitis. 5 pediatric cases without RIF were selected as controls (3 cases were nephrotic syndrome and 2 cases were Henoch-Schonlein purpura nephritis). H&E, Masson, and IHC staining were performed on all patient kidney tissue sections.

### Statistical analysis

All experiments were repeated three times. Statistical analyses were performed using SPSS 22.0 software (SPSS Inc., U.S.A.). All data are presented as mean values ± SD and *p* < 0.05 was considered statistically significant.

## Results

### ILEI facilitates EMT and RIF in the UUO mouse model

**Fig. 1:**
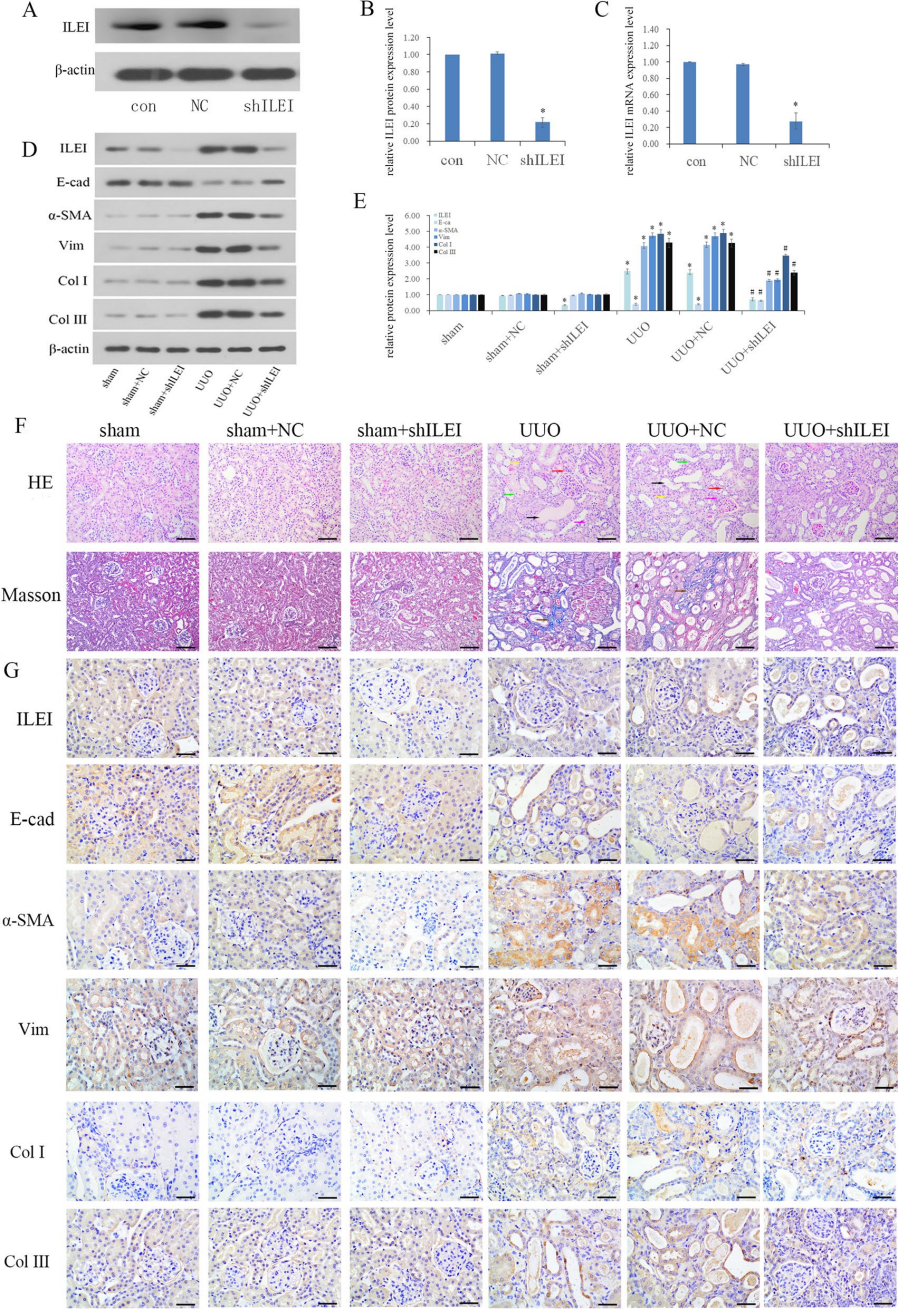
ILEI facilitates EMT and RIF in a mouse UUO model. **A**–**C** WB and real-time PCR analyses of ILEI expression after sham or UUO mice were treated with AAV-ILEI shRNA or AAV-NC shRNA, *p < 0.05. **D**, **E** WB analysis of protein expression of ILEI, E‐cadherin, α‐SMA, vimentin, collagen I, and collagen III, *p < 0.05, ^#^*p* < 0.05. **F** H&E and Masson staining of renal tissue. **G** IHC analyses of ILEI, E‐cadherin, α‐SMA, vimentin, and collagen I and III renal expression

We first verified the effect of shRNA-induced silencing of ILEI in mice of through WB and real-time PCR, showing that ILEI protein and mRNA expression decreased significantly following shRNA injection into the mice (Fig. 1A–C). Mice were divided into six groups: sham, sham with NC, sham with ILEI shRNA treatment, unilateral ureteral obstruction, UUO with NC, and UUO with ILEI shRNA treatment. H&E and Masson staining were performed on kidney tissue from each mouse group.

Kidney tissues were tested by WB. Compared with the sham and sham + NC groups, the UUO and UUO + NC groups had altered EMT and fibrosis markers, with E-cad significantly decreased and increased α-SMA, vim, and collagen I and III. ILEI protein expression also increased, showing that ILEI is involved in UUO-induced EMT. Comparing UUO and UUO + NC with UUO + shILEI showed that the EMT-related indicators all reversed after ILEI silencing, indicating that ILEI plays a catalytic role in EMT (Fig. 1D, E).

From H&E staining, we did not find obvious pathological changes in sham, sham + NC and sham + shILEI groups, compared with these three groups, there were following changes in UUO and UUO + NC: part of the renal tubules atrophied and disappeared (yellow arrow), the distal convoluted tubules and collecting ducts expanded (black arrow), the renal tubular epithelial cells became necrotic (green arrow), and inflammatory cell infiltrated in the renal interstitium (red arrow), there were fibroblast proliferation and fibrosis in the renal interstitium (pink arrow). Moreover, compared with the UUO + NC group, the dilated renal tubules were reduced, the number of renal tubules increased and the shape was close to normal in the visual field, and the interstitial fibrosis and inflammatory cells were significantly reduced in the UUO + shILEI group. Comparing the UUO + shILEI group with the UUO and UUO + NC groups, we found that the blue collagen deposits in the renal interstitial area was significantly reduced after ILEI being knocked down from Masson staining (brown arrow, Fig. 1F).

Through immunohistochemical experiments, we observed that the UUO and UUO + NC groups had decreased E-cadherin; increased α-SMA, vimentin, and collagen I and III; and increased ILEI expression compared with the sham, sham + NC, and sham + shILEI groups. After ILEI silencing in the UUO + shILEI group, E-cadherin increased and α-SMA, vimentin, and collagen I and III all decreased compared to the UUO and UUO + NC groups. Based on our previous research [14], this further reinforces that ILEI is involved in RIF caused by UUO (Fig. 1G).

### ILEI forms a protein complex with LIFR and activates LIFR

**Fig. 2:**
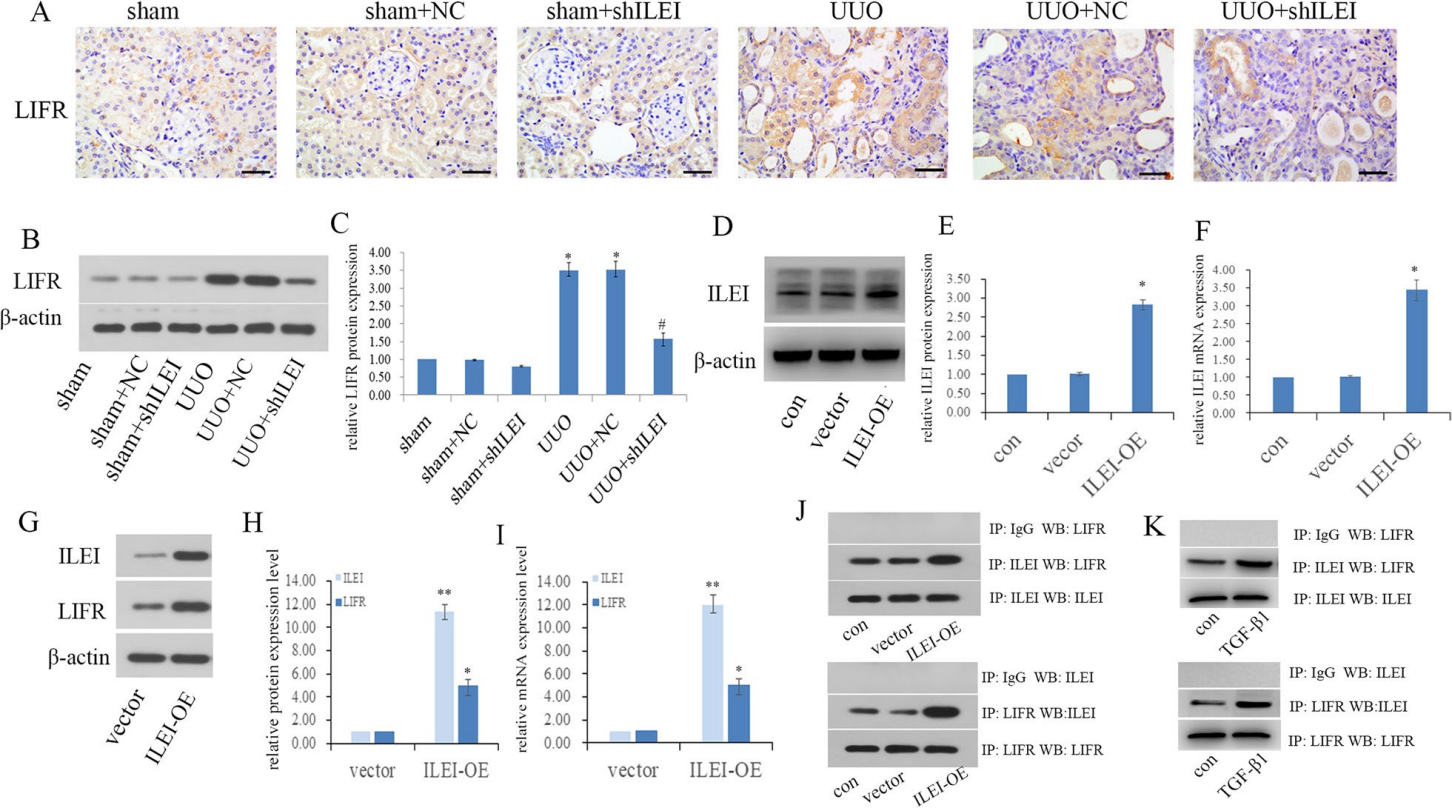
ILEI forms a protein complex with LIFR and activates LIFR **A** IHC analysis of LIFR renal expression in mice. **B**, **C** WB analyses of LIFR renal expression in mice, *p < 0.05. **D**–**F** LIFR overexpression efficiency in HK2 cells examined by WB and real-time PCR, *p < 0.05. **G**–**I** The expression level of LIFR examined by WB and real-time PCR after ILEI overexpression, *p < 0.05, **p < 0.01. **J** Co-IP showing interactions between ILEI and LIFR in ILEI-overexpressed HK2 cells. **K** Co-IP showing interactions between ILEI and LIFR in TGF-β1 induced EMT in HK2 cells

Using IHC staining, we found the UUO and UUO + NC groups had significantly increased expression of LIFR both on the cell membrane and cytoplasm, and this was accompanied by increased ILEI expression. Comparing the UUO and UUO + NC groups with the UUO + shILEI group, we found that LIFR expression also decreased after ILEI was knocked down. These trends were verified via WB (Fig. 2A–C). The results above suggest that LIFR might act as a downstream effector of ILEI.

We validated the relationship between ILEI and LIFR in vitro. First, we overexpressed ILEI and verified the effect by WB and real-time PCR (Fig. 2D–F). Compared to the vector group, ILEI overexpression in the ILEI-OE group was accompanied by increased LIFR at the transcriptional and translational levels (Fig. 2G–I). We used co-IP to explore the interaction between ILEI and LIFR (Fig. 2J) and found that ILEI could bind with LIFR and form a protein complex in HK2 cells and that overexpression of ILEI could upregulate LIFR expression. To verify this conclusion, we performed the co-IP experiment again in the classical cellular model of renal interstitial fibrosis, using 5 ng/ml TGF-β1 to treat HK2 cells for 48 h to induce EMT. Co-IP showed that ILEI interacted with LIFR in TGF-β1 induced EMT in HK2 cells (Fig. 2K).

### LIFR mediates the EMT induced by ILEI in vitro

**Fig. 3:**
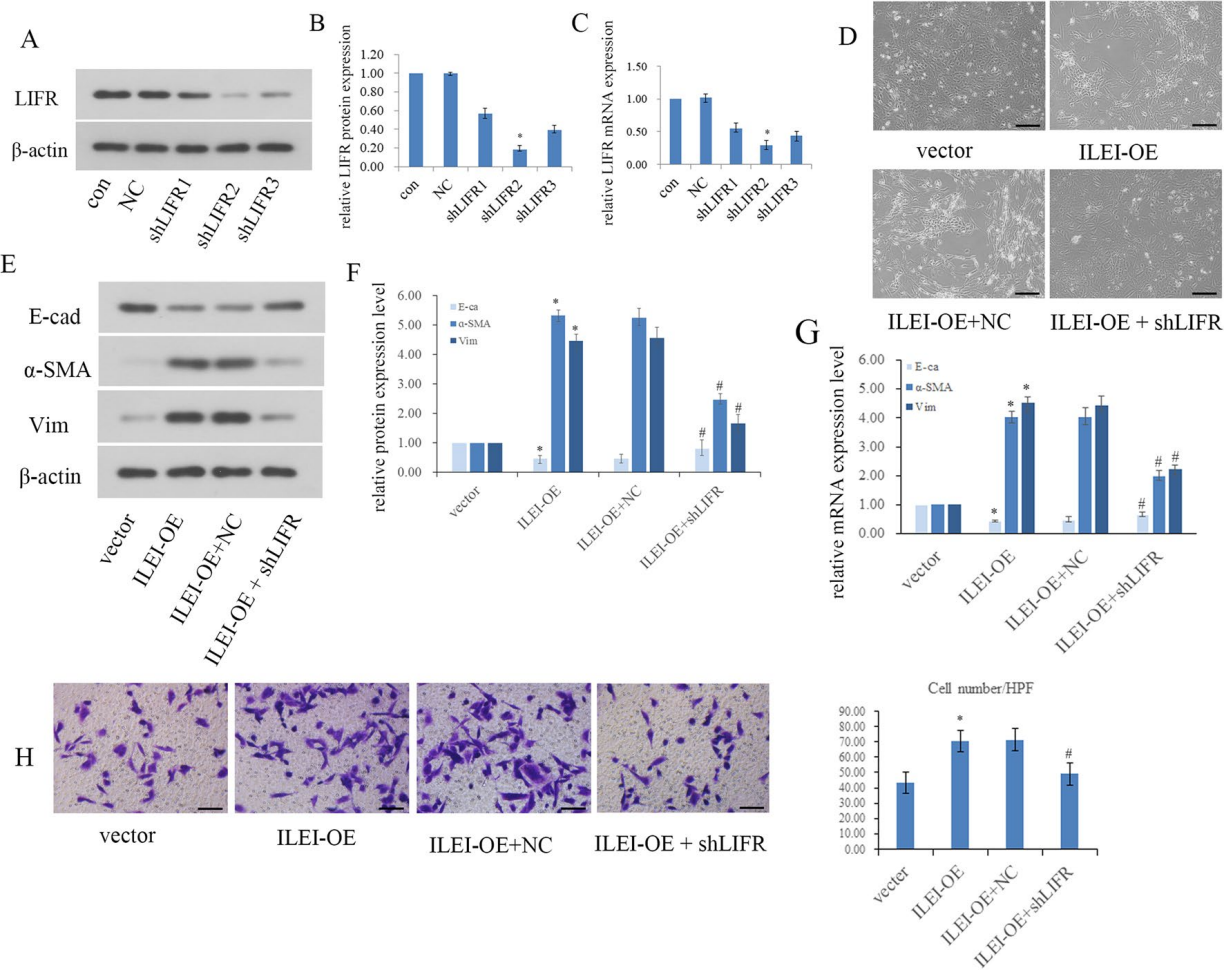
LIFR mediates ILEI-induced EMT in vitro. **A**–**C** LIFR knockdown efficiency in HK2 cells examined by WB and real-time PCR, *p < 0.05. **D** Morphological changes after ILEI overexpression and LIFR knockdown in HK2 cells. **E**–**G** The expression level of E‐cadherin, α‐SMA, and vimentin examined by WB and real-time PCR after LIFR silence in ILEI overexpressed HK2 cells, *p < 0.05, ^#^*p* < 0.05. **H** Transwell assay to evaluate motility changes in HK2 cells*,* *p < 0.05, ^#^*p* < 0.05

Three LIFR‐specific shRNAs were transfected into HK2 cells, with WB and real-time PCR used to identify the one with the best silencing effect (Fig. 3A–C). We confirmed that shLIFR2 had the best silencing at the mRNA and protein levels.

HK2 cells underwent obvious phenotypic changes following ILEI overexpression. Compared to the vector group, ILEI overexpression induced EMT-like morphological changes (Fig. 3D), consistent with our previous study [14]. Overexpression of ILEI decreased E-cadherin expression and increased α-SMA and vimentin at the transcriptional and translational levels, indicating that ILEI is able to induce EMT (Fig. 3E–G). We tested this in transwell assays, finding significantly improved migration ability in HK2 cells after ILEI overexpression (*p* < 0.05), consistent with the results above which is ILEI can induce EMT (Fig. 3H). Therefore, after overexpressing ILEI, EMT was exacerbated in HK2 cells.

We compared the ILEI-OE + NC and ILEI-OE + shLIFR groups and found that silencing LIFR significantly reversed ILEI-induced EMT. After silencing LIFR, HK2 cells regained an oval appearance with close cell–cell adhesions (Fig. 3D). Silencing LIFR also rescued ILEI-induced EMT, characterized by increased E-cadherin expression and decreased expression of α-SMA and vimentin. Real-time PCR showed the same trends at the transcriptional level (Fig. 3E–G). Comparing the ILEI-OE + NC and ILEI-OE + shLIFR groups showed significantly fewer cells passing to the lower chamber upon LIFR silencing, demonstrating that the enhanced cell motility caused by ILEI overexpression was significantly weakened by silencing LIFR (Fig. 3H, p < 0.05). LIFR depletion obviously rescued ILEI-induced EMT, showing that LIFR is a likely downstream factor of ILEI that cooperates with ILEI to promote EMT.

### The ILEI/LIFR complex promotes EMT through the Akt and ERK pathways in vivo and in vitro

**Fig. 4:**
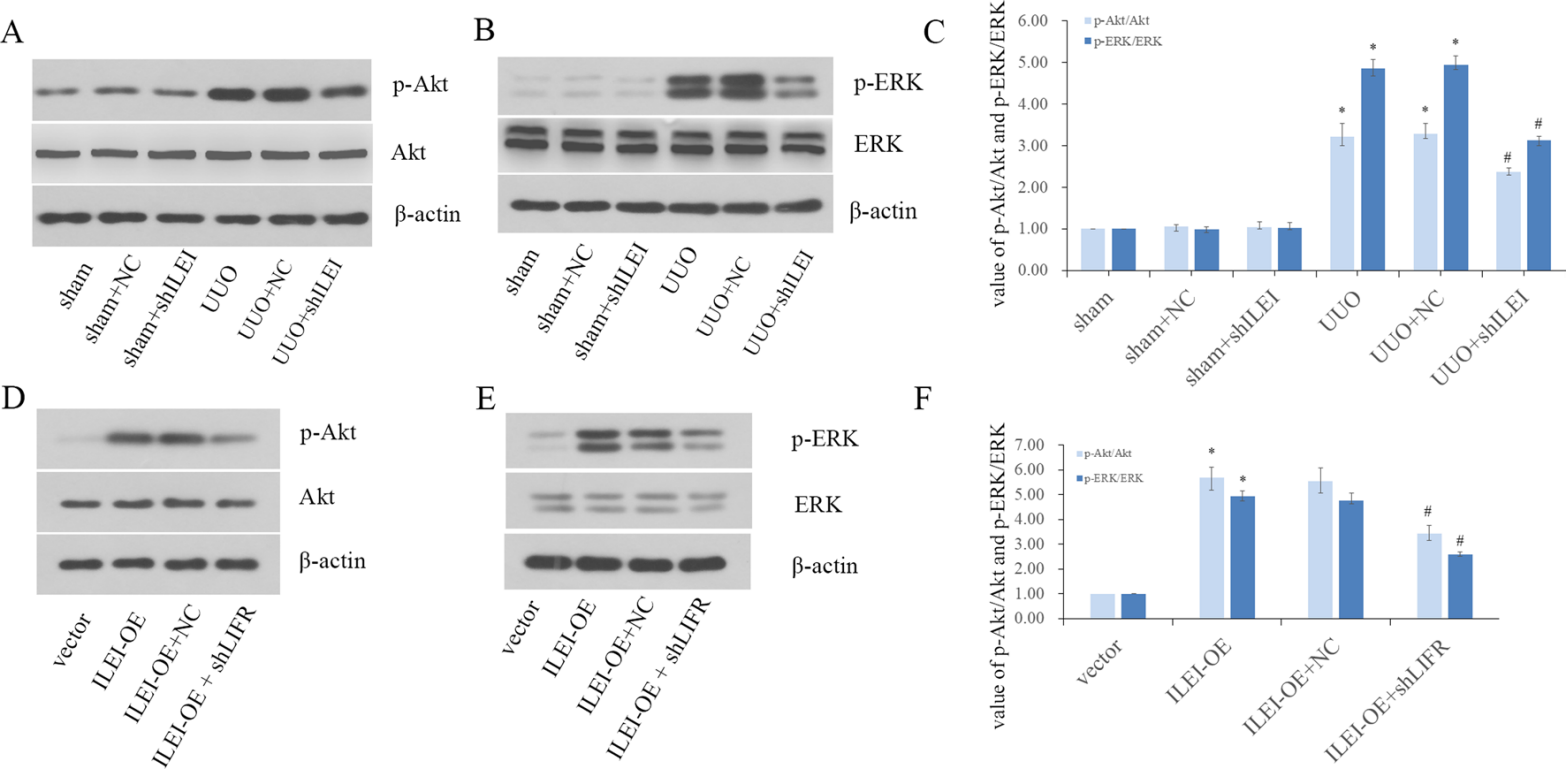
The ILEI/LIFR complex promotes EMT through the Akt and ERK pathways in vivo and in vitro. **A**, **B** WB analysis of Akt, p-Akt and ERK, and p-ERK in vivo. **C** The value of p‐Akt to Akt and p‐ERK to ERK in mice. **D**, **E** WB analysis of Akt, p-Akt and ERK, and p-ERK in vitro. **F** The value of p‐Akt to Akt and p‐ERK to ERK in vitro, *p < 0.05, ^#^*p* < 0.05

We studied the Akt and ERK pathways in vivo. WB analysis showed that total Akt and ERK protein levels remained constant in each group, yet phosphorylation of Akt and ERK increased in all UUO groups, and UUO-induced Akt and ERK phosphorylation were dramatically suppressed upon ILEI knockdown (Fig. 4A–C).

We further tested these effects in vitro. WB analysis of p‐Akt, Akt, p‐ERK and ERK similarly showed that the total amount of Akt and ERK remained constant in each group of HK2 cells, yet phosphorylation of Akt and ERK increased in the ILEI overexpression group. Moreover, this ILEI-induced Akt and ERK phosphorylation was dramatically suppressed when LIFR was knocked down (Fig. 4D–F). These results indicate that the ILEI/LIFR complex promotes EMT through the Akt and ERK pathways.

### ILEI/LIFR contributes to the RIF process in patients

**Fig. 5:**
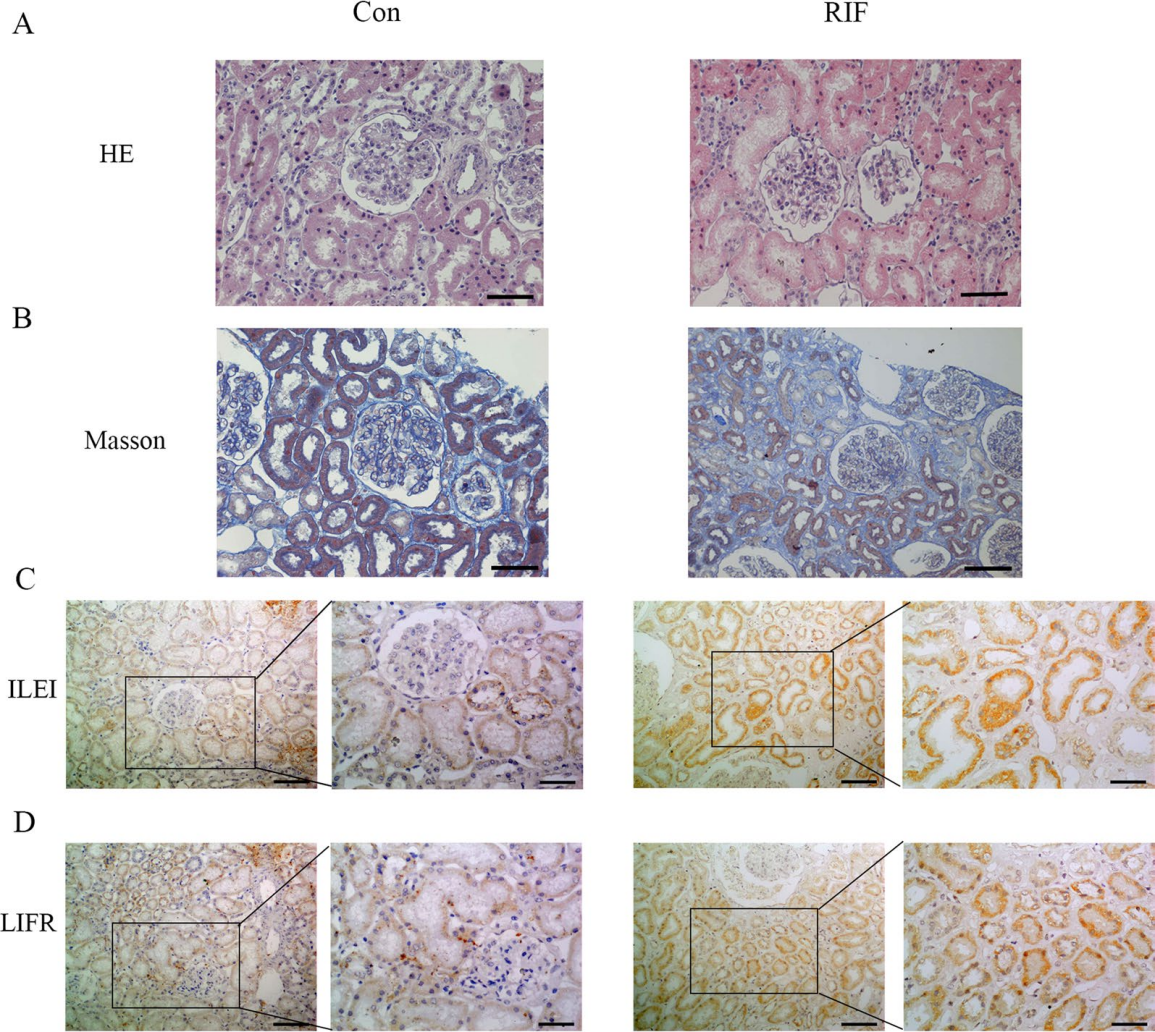
ILEI/LIFR contribute to the RIF process in patients. **A**, **B** H&E and Masson staining of renal tissue from control and RIF patients. **C**, **D** IHC analysis of ILEI and LIFR expression in control and RIF patients

We analyzed 12 pediatric patients with CKD who underwent renal biopsy between February 2017 and May 2021 at the First Hospital of China Medical University, of which 7 were confirmed to have RIF and 5 had no RIF. In the RIF group, renal tubule epithelial cells showed granular degeneration and vacuolar degeneration, renal tubules manifested with focal atrophy and luminal narrowing, and renal interstitial tissues showed edema (Fig. 5A). Masson staining showed obvious blue collagen deposits in the renal interstitial area in the RIF group compared to the non-RIF group (Fig. 5B). ILEI and LIFR were highly expressed on the membrane and in the cytoplasm of renal tubular epithelial cells in the RIF group compared to the non-RIF group (Fig. 5C, D), consistent with ILEI and LIFR contributing to RIF in human patients as well as in the experimental models shown above.

## Discussion

RIF is a widely accepted index of CKD and an important pathophysiological mechanism in ESRD formation. Interstitial accumulation of ECM and related molecules are key characteristics of RIF. Although controversy remains, EMT is considered one of the most important processes leading to interstitial fibrosis. EMT has emerged as a well-regulated process by which epithelial cells lose apical-basal polarity and cell–cell adhesions and acquire mesenchymal characteristics. This transition plays a crucial role in many biological processes including embryonic development, tissue regeneration, wound healing, organ fibrosis, and cancer progression. Numerous studies have shown that EMT is a key factor in three aspects of kidney injury: (1) impacting TEC function, (2) causing cell cycle arrest at the G2 phase, disrupting the balance between repair and fibrosis, (3) immune cell recruitment and inflammation [21, 22]. In this article, we chose E-cadherin, α-SMA, vimentin, collagen Ι and collagen ΙII as important indicators for the detection of EMT and RIF. E-cadherin is an important epithelial cell markers involved in mechanisms regulating cell–cell adhesions, mobility and proliferation of epithelial cells. Alpha-smooth muscle actin (α-SMA) is an excellent marker of myofibroblasts that were recognized as the principal effector cells that are responsible for the excess deposition of interstitial extracellular matrix under pathologic fibrosis conditions [23]. Vimentin is a type III intermediate filament protein that is expressed in mesenchymal cells, which is often used as a marker of mesenchymally-derived cells or cells undergoing EMT [24]. Collagen Ι and collagen ΙII are major components of the extracellular matrix in a variety of organs [25, 26].Therefore, when excessive accumulation of extracellular matrix components leads to fibrotic conditions, collagen Ι and collagen ΙII are important markers in the demonstration of fibrosis [27].

As the gene encoding ILEI, *FAM3C* has been identified as an EMT-specific gene [28] and an emerging biomarker and potential therapeutic target for cancer [29]. Multiple studies have shown that ILEI participates in EMT and promotes metastasis and invasiveness in different tumors [30–32]. Kral et al. showed that ILEI self-assembly into monomers and covalent dimers is essential for EMT induction, tumor growth, and metastasis in cancer cell lines and tumors [33]. LIFR mainly binds with its corresponding ligand leukemia inhibitory factor (LIF) with low affinity and then recruits gp130 to form a heterodimerization complex, then leads to activation of downstream signal, for example the ERK/MAPK and Akt/PI3K pathways, exerting multiple biological functions including EMT and fibrosis. Currently, there are rare studies on the relationship between ILEI and LIFR, Woosley et al. demonstrated that in breast cancer stem cells, ILEI could bind with the extracellular cytokine binding region of LIFR to promote the tyrosine phosphorylation of LIFR, after this transformation, LIFR enhances the phosphorylation of STAT3, further activating Jak/STAT signaling. The above series of changes can maintain breast cancer stem cells tumorigenicity and metastatic potential through EMT [18]. We speculated that LIFR could be involved in the pathological process as a downstream effector molecule of ILEI during RIF.

Akt and ERK are well-defined pathways which are closely associated with fibrosis, and multiple studies have shown that they are hyperactive after phosphorylation and can enhance fibroblast activation, collagen synthesis and EMT process, which will finally lead to fibrotic condition. We have confirmed that ILEI mediates the phosphorylation of Akt and ERK caused by TGF‐β1. Meanwhile, there are studies reported that LIFR activates the JAK/STAT, ERK/MAPK, and Akt/PI3K pathways in tumor formation, inflammation, and cardiac function. So, we supposed LIFR also contribute to the phosphorylation levels of Akt and ERK caused by ILEI.

In our previous study, we demonstrated in vitro that ILEI is a crucial mediator of TGF-β1-induced renal tubular EMT via the Akt and ERK pathways and that ILEI overexpression can independently promote EMT [14]. Based on this previous research, we explored mechanisms by which ILEI may promote EMT in RIF. First, we directly overexpressed ILEI in HK2 cells to drive EMT and found a simultaneous increase in LIFR expression. Knocking down LIFR rescued the EMT caused by ILEI overexpression, thus we conclude that ILEI promotes EMT by stimulating LIFR. The fact that ILEI and LIFR form a protein complex was further proven by immunoprecipitation. We also reveal that ILEI/LIFR promotes EMT through phosphorylation of Akt and ERK. In summary, our in vitro work demonstrates that ILEI binds to LIFR to form a protein complex, activates LIFR, and promotes renal tubular EMT through the Akt and ERK pathways.

We tested the effect of ILEI in vivo using the classic UUO model to cause renal fibrosis. The UUO group presented with RIF and changes in EMT-related markers such as decreased E-cadherin and increased α-SMA and vimentin, showing that EMT is associated with RIF. During this process, increased expression of ILEI and LIFR suggests that these proteins are involved in RIF. After ILEI was knocked down, we found EMT and RIF significantly alleviated and the expression of LIFR reduced. We further compared kidney specimens from patients with or without RIF and found that ILEI and LIFR were both highly expressed in the RIF group. Therefore, we reached consistent conclusions in vivo and in vitro.

Based on our previous study, we mainly conduct in-depth research from the following two aspects: firstly, we construct the mouse model of RIF for validation and make an effort to collect the precious specimens of children with RIF, which has greater clinical predictive significance; secondly, we reasonably speculated and demonstrated that LIFR participates in RIF formation as a downstream effector of ILEI. We have proved for the first time that LIFR is a crucial mediator of renal tubular EMT and RIF. Our research provides a more in-depth study of the molecular mechanisms and regulatory networks of RIF. In future studies, we will strive to uncover the molecular mechanism of the occurrence and development of RIF and explore new therapeutic targets in order to delay the progression of chronic kidney disease.

## Conclusions

In conclusion, we have demonstrated that ILEI is a novel inducer of RIF. Moreover, ILEI forms a protein complex with and activates LIFR, promotes EMT through the Akt and ERK pathways, and regulates RIF aggravation. We hope our observations will guide development of treatments for preventing tubular interstitial fibrosis progression and delay the onset of end‐stage renal failure.

## Data Availability

All data generated or analyzed during this study are included in this published article.
